# Clinical and computed tomography outcome after primary and revisional fundoplication

**DOI:** 10.1007/s00464-026-12760-2

**Published:** 2026-04-02

**Authors:** Julian Süsstrunk, Anna Zurfluh, Daniel Stimpfle, Alexander Wilhelm, Enea Marco Ghielmini, Silke Potthast, Urs Zingg

**Affiliations:** 1 Department of General, Vascular & Visceral Surgery, Centre for Gastro-Esophageal Reflux Disease, Limmattal Hospital, Schlieren, 8952 Zurich, Switzerland; 2 Department of Visceral Surgery, Clarunis University Digestive Health Care Center, St. Clara Hospital and University Hospital, 4002 Basel, Switzerland; 3 Department of Visceral Surgery and Transplantation, University Hospital Zurich, 8091 Zurich, Switzerland; 4 Department of Radiology, Limmattal Hospital, Schlieren, 8952 Zurich, Switzerland

**Keywords:** Revisional surgery, Redo fundoplication, Hiatal hernia repair, Reflux, GERD, Radiological outcome

## Abstract

**Background:**

Failure of laparoscopic fundoplication (LF) occurs in up to 10% and revisional laparoscopic fundoplication (RLF) becomes an option. Data on clinical and anatomical outcomes using computed tomography after RLF are limited.

**Methods:**

All patients undergoing RLF for gastroesophageal reflux disease (GERD) and/or hiatal hernia (HH) between 2015 and 2022 were compared to primary LF-patients (PLF) using a 1:5 weighted propensity score matching. The primary outcome was 1-year recurrence of symptoms (in patients with GERD) or radiological wrap slippage or HH recurrence in patients with HH. Secondary outcomes included the Gastrointestinal Quality of Life Index (GIQLI) and perioperative and postoperative data.

**Results:**

There were 31 RLF (6 GERD, 25 HH) and 120 (36 GERD, 84 HH) PLF cases. Symptomatic recurrence for GERD indication after 1 year was seen in 16.7% after RLF and 11.1% after PLF (*p* = 0.54). Radiological abnormalities in HH were seen in 36% after RLF (24% minor slippages and 12% recurrent hernias) and 15.5% after PLF (11.9% minor slippage and 3.6% recurrent hernias; *p* = 0.028).

Operative time (188 min vs. 147 min, *p* < 0.001) and hospital stay (6.6 days vs. 3.8 days, *p* < 0.001) were significantly longer after RLF. Early complication rates were higher in RLF (16.1% vs. 3.3%, *p* = 0.007). GIQLI scores were comparable (122.6/144 after RLF vs. 124.4/144 after PLF, *p* = 0.58), with > 92% of patients in both groups reporting good/excellent outcomes.

**Conclusion:**

RLF is associated with longer operative time, increased morbidity, and higher rates of CT abnormalities compared to PLF. Symptom control and quality of life were comparable.

**Graphical abstract:**

**Figure:**
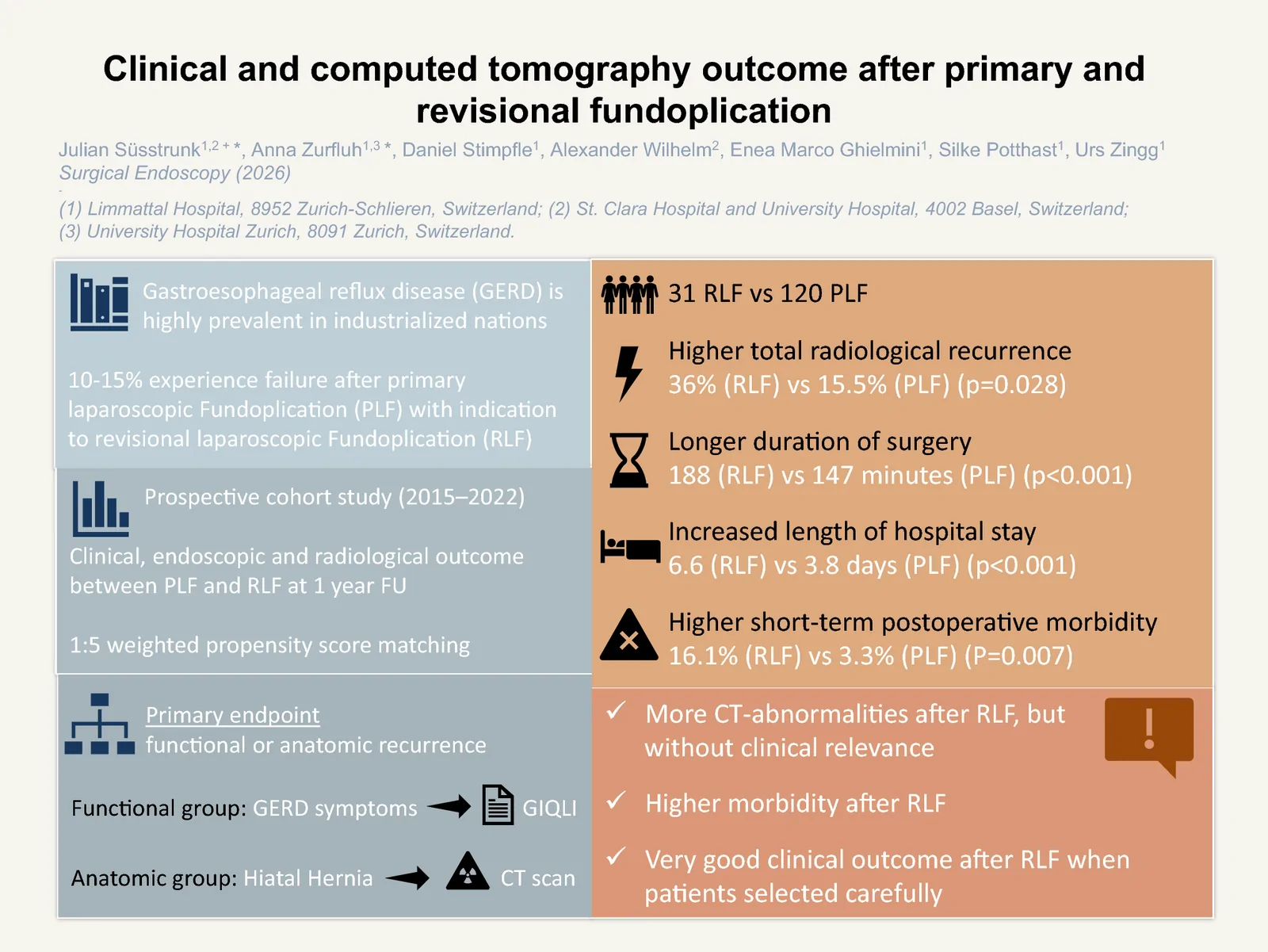

**Supplementary Information:**

The online version of this article (https://doi.org/10.1007/s00464-026-12760-2) contains supplementary material, which is available to authorized users.

Gastroesophageal reflux disease (GERD) is highly prevalent in industrialized nations. In patients with unsatisfying symptom control using proton pump inhibitors (PPI), volume reflux symptoms, development of Barrett’s esophagus or with relevant anatomical abnormalities such as a hiatus hernia (HH), hiatal repair, and laparoscopic fundoplication (LF) are treatment options with excellent clinical outcomes [1, 2]. Although the success rate is high, a minority of patients experience failure of LF and re-operation may be indicated in 3–10% of the cases [3–7].

The underlying causes for LF failure or failure of the hiatus hernia repair and the subsequent recurrence of symptoms are numerous and manifold [8]. Systematic diagnostic assessment with endoscopic, functional and radiological examination is therefore necessary to identify anatomical abnormalities and functional disorders to select patients who might benefit from a revisional laparoscopic fundoplication (RLF).

There is a variety of methods for RLF for recurrent HH or GERD, with takedown of the initial wrap, hiatal repair with or without a mesh and refashioning of a new total or partial wrap being nowadays the most common approach [9]. Outcomes after RLF have improved in the last decades, a recent systematic review and meta-analysis reported clinical success rates ranging from 57 to 94%, based on registration of symptom recurrence [10]. However, in most studies the technique is heterogeneous and objective outcome data after RLF repair using systematic clinical and especially radiological follow-up is lacking. Thus, the aim of this study is to investigate the clinical and radiological outcomes after RLF, using a standardized technique, in comparison to a matched cohort of patients undergoing primary laparoscopic fundoplication (PLF).

## Patients and methods

### Study design and patient selection

This is a prospective single center cohort study of patients who had undergone RLF and PLF for GERD and/or HH between 2015 and 2022. The study protocol was approved by the local ethics committee (ZHEK BASEC 2016-01510) and registered on clinicaltrials.gov (NCT03025932). Written informed consent was obtained from each participant after full explanation of the purpose and nature of each intervention.

**Fig. 1:**
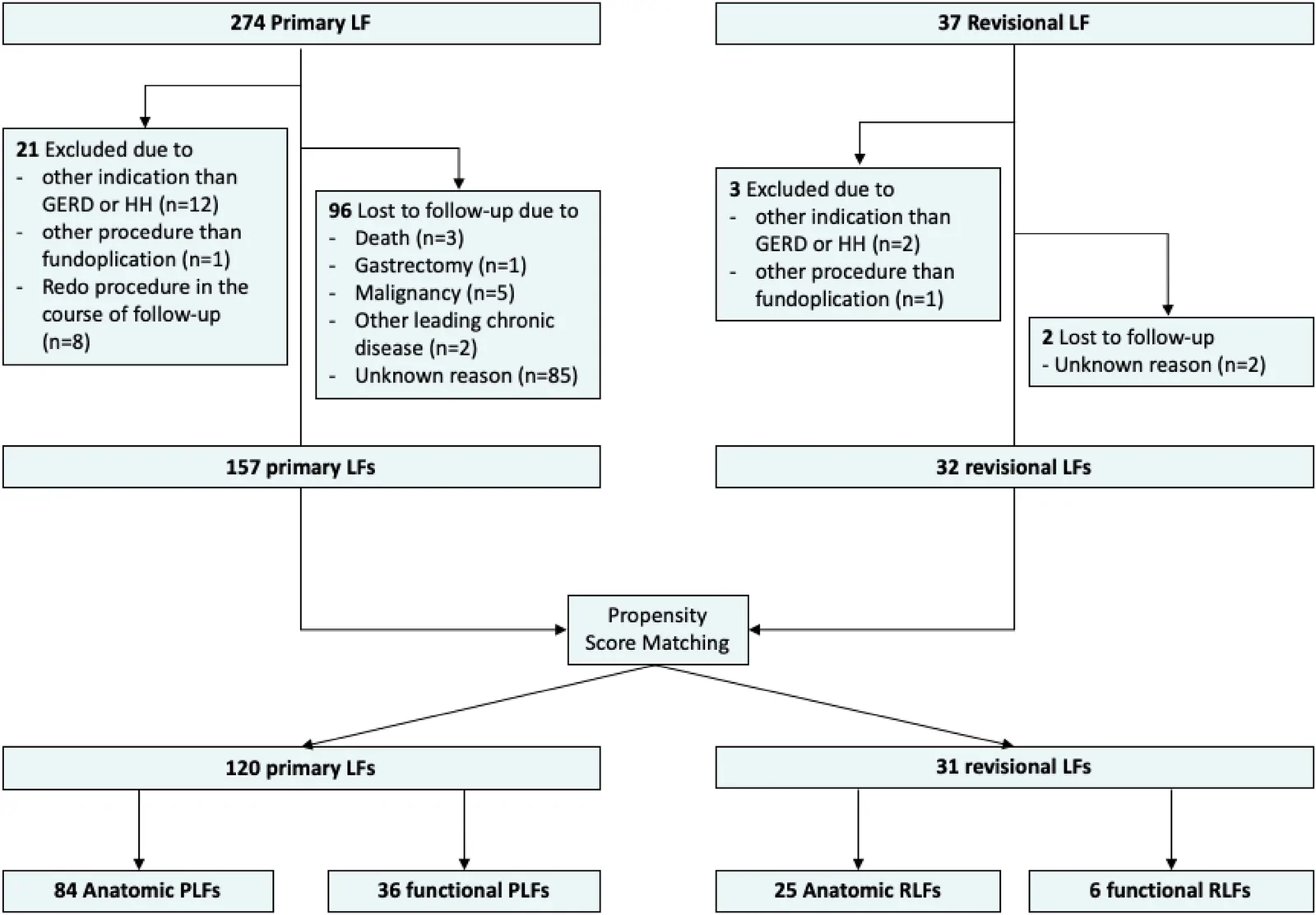
Patient selection. *GERD* gastroesophageal reflux disease, *HH* hiatal hernia, *LF* laparoscopic fundoplication, *PLF* primary laparoscopic fundoplication, *RLF* revisional laparoscopic fundoplication

Selection process is shown in Fig. 1. Patients were only included if the indication for fundoplication was GERD and/or HH. Patients without clinical follow-up (patients with GERD) or radiological follow-up (patients with HH) after 1 year were excluded (5.8% in RLF and 33.5% in PLF, respectively). Patients that were initially in the primary group were moved to the RLF group if they underwent a revisional operation.

### Preoperative assessment

Preoperative workup included a thorough investigation of medical history, clinical examination, and preoperative upper endoscopy for all patients. In patients with GERD or recurrence of GERD an additional functional workup was performed including high-resolution esophageal manometry (HRM) and pH-studies (pH impedance or wireless pH testing). The diagnosis was made according to the Lyon consensus of 2017 [11]. In patients with esophagitis grade C & D according to the Los Angeles classification or long-segment Barrett's esophagus, no pH-study was planned. In patients with endoscopically large HH (>5 cm) and in all patients planned for RLF a preoperative computed tomography (CT) with oral and intravenous contrast was performed.

### Definitions, indication, and operative procedure

Hiatal hernia and GERD are often used synonymously without further differentiation. However, this does not often mirror the clinical status as, for example, patients with large hernias or upside-down stomach often present with other symptoms than classical GERD, such as retrosternal pain, dyspnea and dysphagia or coughing and concomitant presence of a HH can increase the severity of reflux-related symptoms [12, 13]. Thus, we grouped patients in functional and anatomic. Functional was defined as GERD with or without a concomitant hiatal hernia of maximal 5 cm. The surgery was recommended if medical therapy proved unsuccessful, volume reflux was present, or according to patients’ preference.

In the anatomical group, the indication for primary surgery was either the CT-demonstrated herniation of >20% of stomach into the chest cavity in symptomatic patients or >50% herniation in oligosymptomatic patients.

In the revisional cohort the aforementioned differentiation was also used. Thus, the indication was either recurrence of GERD, diagnosed as described above, or symptomatic herniation of the fundoplication wrap or stomach into the thoracic cavity.

All PLF and RLF were performed by a standardized technique using five laparoscopic ports by or under the supervision of one surgeon (UZ).

In PLF, the first step was the preparation of the right and left pillar of the right crus with identification and preservation of vagal nerves. A present hernia sac and its contents were reduced from the mediastinum into the abdominal cavity. The esophagus was mobilized to reach an intraabdominal length of at least 2 cm below the hiatus to allow tension-free positioning. No standardized division of short gastric vessels was done. A dorsal hiatoplasty using nonabsorbable sutures was performed and calibrated with a 52Fr-Maloney bougie. In patients with an anatomical indication, the hiatoplasty was reinforced with a 3×4 cm posterior polypropylene mesh. The anti-reflux part consisted of the reconstruction of the angle of His with two nonabsorbable sutures and a 180° anterior fundoplication as described by Watson et al. [14]. In patients with functional indication, no mesh was used and a posterior 270° wrap according to Toupet was fashioned with 11 nonabsorbable sutures.

In RLF, the first step was the takedown of all adhesions. The hernia sac and its contents including a migrated wrap were reduced into the abdominal cavity. In all patients, the primary fundoplication wrap was opened completely to restore the normal anatomy. In all cases, an intraoperative upper endoscopy was performed to assess the result of the takedown and to check for any injuries to the wrap, esophagus, or stomach. Then, the reconstruction and wrap were performed identical to the PLF procedures.

### Postoperative care and follow-up

Postoperative care included a contrast study on day 1 to check for leaks and to analyze the postoperative passage. Patients were advised to stay for 2 weeks each on minced and soft diet. The first clinical follow-up was conducted after 6 weeks. Upper endoscopy was routinely performed in all patients 3 months postoperatively to confirm the wrap position, exclude postoperative complications and assess the evolution of reflux-associated pathologies. All patients with an anatomical indication in the PLF as well as in all patients undergoing RLF a routine CT scan with oral contrast at 1-year follow-up was performed. Patients undergoing PLF for functional indication in absence of HH did not receive a CT scan at 1-year follow-up.

### Outcome measures

The primary endpoint was overall recurrence of the initial medical problem, defined for the functional group as a subjective recurrence of symptoms with a numeric rating scale (NRS 1–10 points, 1 representing no symptoms, 10 representing the worst possible symptoms) of >3 points for retrosternal burning or reflux symptoms, and for the anatomical group proven CT-radiographic recurrence of HH. As the CT scan allows for a very detailed visual assessment, the type of wrap migration was differentiated into slippage (less than 3 cm of wrap migration cephalad of the diaphragm) and true recurrence (more than 3 cm of wrap migration) [15, 16]. CT scans were analyzed independently by an upper GI surgeon and a gastrointestinal radiologist and in case of discrepant findings, consent was reached.

Secondary endpoints included clinical outcome assessed for various symptoms such as reflux, retrosternal burning, flatulence, epigastric pain, and bloating using the NRS, Quality of life (QoL) assessed using the validated Gastrointestinal Quality of Life Index (GIQLI), and postoperative endoscopic findings [17].

### Statistical analysis

The baseline characteristics and outcome parameters were compared between the treatment groups with a two-sample *t* test for continuous data and a Pearson’s *χ*^2^ test for categorical data. Continuous data are presented as mean ± standard deviation (SD). Categorical data are summarized using frequencies and proportions.

To reduce confounding, propensity score matching (PSM) was performed using a nearest neighbor matching algorithm. The propensity score was estimated via logistic regression including the covariates sex, age, main indication (GERD or HH), and body mass index (BMI). As the incidence of Barrett’s esophagus was low, motility disorders were not routinely assessed preoperatively and prior mesh use was an exclusion criterion, these criteria were not suitable for matching criteria. Furthermore, despite hernia size/type being a relevant matching criterion, in the matched cohort those criteria were not statistically significantly different, and we therefore do not see any added value in adding those matching factors additionally. Patients in the RLF group were matched to controls in a 1:5 ratio without replacement. A caliper width of 0.2 standard deviations of the logit of the propensity score was applied to restrict poor matches. Covariate balance before and after matching was assessed using standardized mean differences (SMD).

A *p*-value of 0.05 was considered significant. Statistical analyses were performed using the R statistical package (R Studio Version 2023 V4.3.1, www.r-project.org).

## Results

### Patient characteristics

After patient selection using a 1:5 weighted propensity score matching, 31 RLF procedures in 29 patients and 120 PLF procedures/patients were included in the analysis (Fig. 1). Indication for RLF was anatomic recurrence in 25 cases and functional recurrence in 6 cases. Primary procedures were performed for anatomic indication in 84 patients and for functional indication in 36 patients. Demographic parameters were comparable across the two groups as summarized in Table 1.

**Table 1:** Patient characteristics of patients undergoing primary fundoplication (PLF) and revisional fundoplication (RLF)

	PLF (*n* = 120)	RLF (*n* = 31)	*p*-Value
Gender			0.73
Female	70 (58.3%)	17 (54.8%)	
Male	50 (41.7%)	14 (45.2%)	
Age at operation, mean (SD)	65.73 (13.97)	65.39 (13.42)	0.90
BMI, mean (SD)	28.23 (4.82)	28.71 (4.19)	0.62
ASA score			0.70
1	8 (6.7%)	2 (6.5%)	
2	81 (67.5%)	24 (77.4%)	
3	30 (25.0%)	5 (16.1%)	
4	1 (0.8%)	0 (0.0%)	
Main indication			0.24
Functional	36 (30.0%)	6 (19.4%)	
Anatomic	84 (70.0%)	25 (80.6%)	
GERD type			0.29
No GERD	57 (47.5%)	13 (41.9%)	
Classic reflux	32 (26.7%)	14 (45.2%)	
Volume reflux	8 (6.7%)	1 (3.2%)	
Atypical	3 (2.5%)	0 (0%)	
Combined	20 (16.7%)	3 (9.7%)	
Preoperative medication for GERD			0.47
None	23 (19.2%)	8 (25.8%)	
PPI in low dose	57 (47.5%)	16 (51.6%)	
PPI in high dose	40 (33.3%)	7 (22.6%)	
Type of hiatal hernia			0.088
No hiatal hernia	10 (8.3%)	4 (12.9%)	
Axial	36 (30.0%)	4 (12.9%)	
Pure para-esophageal	1 (0.8%)	1 (3.2%)	
Mixed	49 (40.8%)	19 (61.3%)	
Upside down stomach	24 (20.0%)	3 (9.7%)	
Extent classified			0.15
No hiatal hernia	10 (8.3%)	4 (12.9%)	
Small (< 20% of stomach)	38 (31.7%)	5 (16.1%)	
Medium (21–50% of stomach)	40 (33.3%)	16 (51.6%)	
Large (> 50% of stomach)	32 (26.7%)	6 (19.4%)	

*SD* standard deviation, *BMI* body mass index (kg/m^2^), *ASA Score* American Society of Anesthesiologists Score, *GERD* gastroesophageal reflux disease, *PPI* proton pump inhibitor

### Perioperative data

Hiatoplasty and anterior 180° fundoplication with reconstruction of the angle of His was the most commonly performed procedure in both groups, with 83.9% of patients in the RLF group (26/31) and 80.8% in the PLF group (97/120).

In RLF, the mean time between the initial and the revisional procedure was 69.8 ± 58.9 months. Two patients required multiple reoperations; one of these patients subsequently underwent a total gastrectomy 6 years and 5 months after the last redo fundoplication due to persistent, uncontrollable dysphagia, pain, and delayed gastric emptying.

There was a significant difference in mean duration of surgery with 188 min in RLF and 147 min in PLF (*p*<0.001). Length of stay was significantly higher after RLF with 6.6 days compared to 3.8 days after PLF (*p*<0.001). The detailed perioperative data are summarized in Table 2.

**Table 2:** Perioperative data of patients undergoing primary fundoplication (PLF) and revisional fundoplication (RLF)

	PLF (*n* = 120)	RLF (*n* = 31)	*p*-Value
Number of redo procedures			
Operation			0.70
270° posterior	23 (19.2%)	5 (16.1%)	
180° anterior	97 (80.8%)	26 (83.9%)	
Duration of surgery (min), mean (SD)	146.45 (40.35)	187.58 (67.20)	< 0.001
Access			0.048
Laparoscopic	120 (100%)	30 (96.8%)	
Conversion	0 (0%)	1 (3.2%)	
Mesh reinforced hiatoplasty			0.24
No	36 (30.0%)	6 (19.4%)	
Yes	84 (70.0%)	25 (80.6%)	
Length of stay after operation (days), mean (SD)	3.8 (2.39)	6.6 (5.66)	< 0.001
Reoperation within 30 days			0.417
No	119 (99.2%)	30 (96.8%)	
Yes	1 (0.8%)	1 (3.2%)	
Operation-related mortality			1
No	120 (100%)	30 (100%)	
Intraoperative morbidity			
No	114 (95%)	31 (100%)	0.206
Yes	6 (5%)	0 (0%)	
Postoperative morbidity (< 30 days)			0.007
No	116 (96.7%)	26 (83.9%)	
Yes	4 (3.3%)	5 (16.1%)	
Postoperative morbidity (> 30 days)			
No	119 (99%)	30 (97%)	0.37
Yes	1 (1%)	1 (3%)	

*SD* standard deviation

### Postoperative morbidity and mortality

There was no perioperative or 90-day mortality in either group. The overall incidence of early postoperative complications within 30 days after surgery was 16.1% in the RLF group and 3.3% in the PLF group (*p*=0.007). The reoperation rate within 30 days (early reoperation) was comparable in both groups (*n*=1, 3.2% vs. *n*=1, 0.8%; *p*=0.417). In the PLF group, the indication for early (<30 days) and late (>30 days) reoperation included one gastric perforation and one mediastinal hematoma, respectively. In the RLF group, one subphrenic abscess due to gastric perforation led to subsequent surgical revision. Detailed information on type and severity of complications is displayed in Table 3.

**Table 3:** Early and late onset postoperative complications of patients undergoing primary fundoplication (PLF) and revisional fundoplication (RLF)

Clavien–Dindo	Complications after PLF	Onset^a^	Complications after RLF	Onset^a^
2	Urinary tract infection	Early	Urinary tract infection	Early
	Cardiopulmonary decompensation	Early	Mediastinal seroma	Early
	Pulmonary embolism	Early	Pneumonia	Early
3a	Meat bolus impaction	Late		
3b	Small bowel ischemia due to HIT	Early	Gastric perforation, subphrenic abscess	Early
			Mediastinal hematoma, bolus distal esophagus	Early
			Mesh penetration	Late

^a^Early = < 30 days postoperative; late = > 30 days postoperative

*GERD* gastroesophageal reflux disease

### Endoscopic outcomes

The endoscopic results are shown in Table 4. All patients with one exception in the PLF group (patient’s refusal) had a preoperative upper endoscopy, with erosive esophagitis being the most common pathologic finding. Los Angeles grade A esophagitis was observed in 8 patients (6.7%) in the PLF group, while grade B was found in 15.1% of patients in the RLF and 14.3% in the PLF group. Los Angeles grade C or D were present in 4 patients (14.3%) in the RLF group and in 7 patients in the PLF group (5.9%). Barrett’s esophagus was highly prevalent with 19.4% in the RLF and 18.3% in the PLF group, respectively.

**Table 4:** Pre- and postoperative endoscopic results of patients undergoing primary fundoplication (PLF) and revisional fundoplication (RLF)

	PLF (*n* = 120)	RLF (*n* = 31)	*p*-Value
Preoperative endoscopic findings			
GERD	54 (45.0%)	10 (32.3%)	0.20
Cameron’s lesions	24 (20.0%)	2 (6.5%)	0.075
Ulcer	2 (1.7%)	1 (3.2%)	0.58
Barrett esophagus	22 (18.3%)	6 (19.4%)	0.90
Los Angeles classification			0.53
0	71 (59.7%)	17 (60.7%)	
A	8 (6.7%)	0 (0%)	
B	18 (15.1%)	4 (14.3%)	
C	5 (4.2%)	3 (10.7%)	
D	2 (1.7%)	1 (3.6%)	
Not defined	15 (12.6%)	3 (10.7%)	
New pathologic endoscopic findings postoperative			0.068
No	117 (97.5%)	28 (90.3%)	
Yes	3 (2.5%)	3 (9.7%)	

Early =  < 30 days postoperative, late =  > 30 days postoperative

*HIT* heparin-induced thrombocytopenia

Upper endoscopy three months postoperatively showed correct position of the wrap in all RLF patients and all but one patient in the PLF group, this one with an early paraesophageal slipping.

Three patients in each group exhibited new postoperative pathological findings. In RLF, one patient, with a history of two prior extra-institutional procedures (Nissen fundoplication, revisional Nissen fundoplication), had a mesh penetration after the third revisional surgery into the esophagus, which resulted in another revisional procedure. This patient underwent 6 years later the aforementioned gastrectomy. One patient developed a candidiasis and one patient an ulcer, both treated conservatively. In PLF, two patients presented with new onset mild esophagitis and another one with a candidiasis. All of them were treated conservatively. No patient required endoscopic dilatation for stenosis in either group.

### Clinical outcomes

**Fig. 2:**
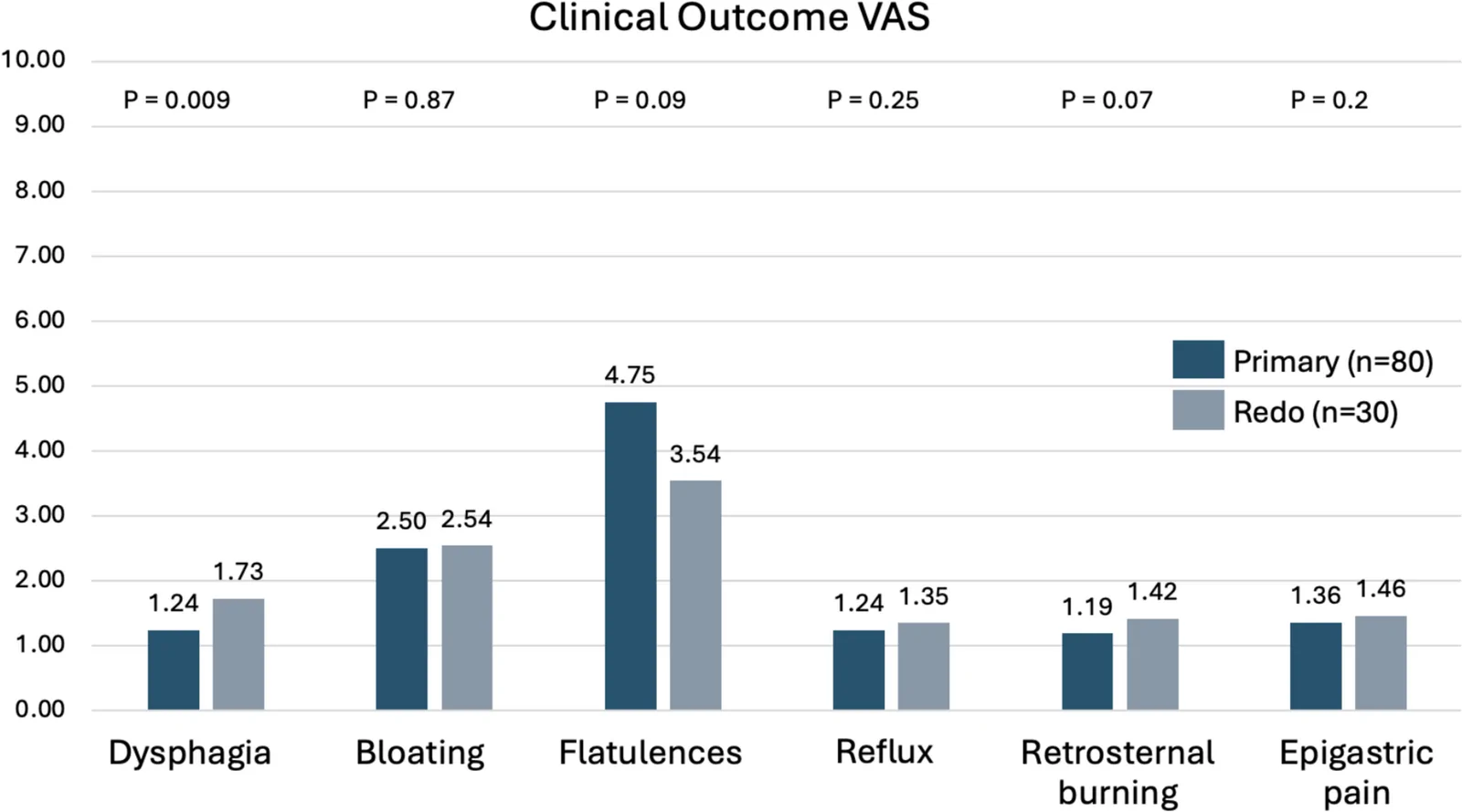
Follow-up questionnaire VAS (visual analog scale with 1 no symptoms and 10 most severe symptoms)

At 1-year follow-up, no significant difference in overall subjective self-reported outcome, rated as good or excellent at time of follow-up, was seen (92.9% in RLF and 95.4% in PLF, *p*=0.34) and the GIQLI score was comparable between groups (122.6/144 in RLF and 124.4/144 in PLF, *p* = 0.58). The discontinuation rate of PPIs 1 year after RLF and PLF was comparable (82.8% vs. 90.1%; *p*=0.33). Follow-up questionnaire results are illustrated in Fig. 2. A significant difference was seen in dysphagia with slightly higher scores after RLF (1.82) compared to PLF (1.27; *p*=0.009), while other GERD-related symptoms showed no significant difference.

### Radiological outcomes

The CT scan 1 year postoperatively showed radiological abnormalities in 36% after RL and 15.5% after PLF (*p*=0.028). Of these, a minor slippage (<3 cm of wrap migration) was seen in 6 (24%) patients after RLF and in 10 (11.9%) patients after PLF (*p* = 0.19) and a true recurrent HH occurred in 3 (12%) patients after RLF and in 3 (3.6%) patients after PLF (*p* = 0.17). All patients with slipping, irrespective of the group, had a good or excellent clinical outcome at 1-year follow-up. Of all patients with a recurrent HH confirmed by CT scan, all had an excellent clinical outcome in both groups and no NRS score >3 was present for any of the symptoms listed above. Detailed outcome parameters are shown in Table 5.

**Table 5:** Clinical and radiological outcome of patients undergoing primary fundoplication (PLF) and revisional fundoplication (RLF)

	PLF (*n* = 120)	RLF (*n* = 31)	*p*-Value
Clinical result 1 year	*n* = 109	*n* = 28	0.34
Excellent	94 (86.2%)	21 (75%)	
Good	10 (9.2%)	5 (17.9%)	
Fair	5 (4.6%)	2 (7.1%)	
Medication for GERD needed	*n* = 111	*n* = 29	0.33
No	100 (90.1%)	24 (82.8%)	
Less than preoperatively	8 (7.2%)	5 (17.2%)	
Equal to preoperative	2 (1.8%)	0 (0.0%)	
More than preoperative	1 (0.9%)	0 (0.0%)	
GIQLI 1 year, mean (SD)	124.42 (19.05)	122.64 (18.23)	0.58
Follow-up questionnaire			
Dysphagia, mean (SD)	1.27 (0.84)	1.82 (1.47)	0.009
Bloating, mean (SD)	2.53 (2.26)	2.61 (2.11)	0.87
Flatulence, mean (SD)	4.44 (2.46)	3.57 (2.17)	0.09
Reflux, mean (SD)	1.22 (0.82)	1.46 (1.50)	0.25
Retrosternal burning, mean (SD)	1.22 (0.61)	1.54 (1.32)	0.07
Epigastric pain, mean (SD)	1.28 (1.00)	1.57 (1.35)	0.20
Would perform surgery again			0.52
No	1 (1%)	0 (0%)	
Yes	87 (99%)	26 (100%)	
Radiological outcome 1 year (CT scan)		0.17	
No data^a^	36	6	
Correct position	71/84 (84.5%)	16/25 (64%)	
Slippage	10/84 (11.9%)	6/25 (24%)	
Recurrent hernia	3/84 (3.6%)	3/25 (12%)	

*GERD* gastroesophageal reflux disease, *GIQLI* gastrointestinal Quality of Life Index, *SD* standard deviation

^a^Patients with primary surgery for GERD with less than 20% migration of stomach into the thoracic cavity were not routinely followed-up with a CT scan

## Discussion

Despite technical challenges, RLF is considered the standard procedure for recurrent symptoms after primary hiatal hernia repair and fundoplication in the current literature [18–25]. As seen in previous studies, however, RLF has been associated with longer operative time, higher morbidity, and a higher probability of failure compared to PLF, similar to our findings. Click or tap here to enter text [26–28]. The present analysis shows a significantly higher radiologic recurrence rate in RLF compared to patients undergoing primary surgery. Despite this higher objective recurrence rate, patient-reported outcome demonstrates symptomatic relief after RLF in nearly all patients at 1-year follow-up, comparable to the PLF group. Furthermore, our outcomes after RLF are comparable to those reported in the literature for primary antireflux surgery [4, 29, 30]. The clinical outcome does not seem to depend on the presence of anatomical recurrence as defined for this study, and clinical benefits of RLF seem to outweigh the risk of recurrence.

Most published studies focused on patient-reported outcomes and quality of life [31, 32]. Additional endoscopic or radiological examinations were only performed if the subjective outcome was not as expected, i.e., patients reported new onset or persisting symptoms. However, anatomical recurrence and symptoms do often not correlate [15, 33, 34]. The present study additionally used a CT scan to evaluate the location of the wrap and the stomach in order to objectively assess anatomical results. The CT allows for a detailed analysis, and we differentiated between a minor slippage less than 3 cm and a full recurrence. Identifying anatomic relapse early may be beneficial to monitor progression either with regular clinical follow-up for symptom assessment or with further CT follow-up. It is, however, important to mention that further data are needed to assess the potential benefit of CT follow-up in detecting complications after LF especially in light of radiation exposure and additional cost of this exam with currently unknown clinical relevance. Our institution currently favors an imaging-based follow-up strategy after fundoplication, especially in patients undergoing surgery for large HH in which the recurrence rate is high [35, 36]. This allows informed decision making on potential additional diagnostics or reintervention if symptoms reoccur. Furthermore, the missing correlation between anatomic recurrence and reappearance of symptoms in most patients indicates that such wrap migrations would have not been recognized if the follow-up were based on symptom questionnaires only.

The anterior wrap is the institution's preference for the repair of HH due to a low rate of postoperative dysphagia, good reflux control, and previously published satisfying radiologic and clinical outcomes from our institution [15]. Furthermore, randomized controlled trials and long-term follow-up studies have demonstrated comparable reflux control and quality-of-life outcomes between anterior partial and other wrap techniques, while anterior fundoplication may be associated with lower rates of postoperative dysphagia and gas-related symptoms [14, 26]. Its less obstructive configuration is particularly attractive in revisional settings, where minimizing outflow resistance and postoperative functional complaints is desirable.

Despite the higher radiologic recurrence in the RLF group, clinical outcomes were excellent and comparable between revisional and primary procedures. At 1 year, improvements in GERD-related symptoms and quality-of-life scores were comparable in both groups. Both RLF and PLF patients diagnosed with recurrent hernia remained highly satisfied with their outcomes. As seen in the postoperative course, dysphagia rates were low in RLF and PLF, and although there was a difference in dysphagia between groups, this is likely not clinically relevant.

Revisional fundoplication is a more complex procedure compared to primary surgery. The adhesiolysis and the complete take-down of the primary wrap explains the longer operative time and resulted also in an increased length of hospital stay, which is generally higher compared to studies from other countries performing minimally-invasive LF, likely linked to the reimbursement system in Switzerland where a short length is stay is not necessarily incentivized [10, 18–20]. Also, we observed a significantly higher morbidity in the RLF group. However, severe complications requiring a reoperation were not different between the groups. Overall, 30-day postoperative morbidity was acceptable and although it was significantly higher after RLF, this is still comparable to current literature [23]. A recently published multicenter retrospective study including over 4000 patients showed overall postoperative morbidity of 13.3%, identifying giant HH and RLF as risk factors for postoperative complications [37].

Some limitations merit mentioning. The number of patients in the RLF group is rather small and data were collected from a single center, which limits the general applicability of the findings. However, there are not many reports with substantially higher numbers of RLF patients [18–23]. One strength of the current study is the complete follow-up of 100% in both groups at 1 year. Furthermore, the use of the CT in all RLF patients as well as in all patients with large hiatal hernias increases the objectivity of this follow-up. Most follow-up studies do not use radiological examinations at all or only in symptomatic patients. This leads to an underestimation of the true recurrence rate. The differentiation between CT-radiological slipping and a higher degree of recurrence is not a standard, as CT scans are not widely used to analyze the postoperative outcome. Still, it allows a detailed assessment and might lead to further investigations or follow-up appointments. Patients undergoing PLF for GERD without large HH were not routinely examined by CT scan and a meaningful statistical analysis in these patients undergoing Toupet fundoplication was not possible due to low numbers, this may be seen as another limitation. However, the wrap migration rate in these patients is known to be low [38]. In case of recurrent symptoms, a CT scan was always obtained to rule out an anatomical problem. All patients with a functional indication for RLF underwent a preoperative CT scan, and no relevant migration of the wrap or stomach was seen. Despite these limitations, next to the complete follow-up a key strength of our study is the use of propensity score matching, which helped minimize confounding and improve the validity of the comparison between the groups.

## Conclusion

Revisional fundoplication has a higher rate of CT abnormalities 1 year postoperative compared to PLF but results in good self-reported subjective outcomes with high patient satisfaction. Despite a longer operation time and hospital stay, and a higher perioperative morbidity, revisional fundoplication should be offered in carefully selected patients as the treatment choice for recurrent symptoms or migration of the wrap.

## Supplementary Information

Below is the link to the electronic supplementary material.

(DOCX 18 kb)
