# Supplementary material for: Clinical and computed tomography outcome after primary and revisional fundoplication
Source: Surg Endosc. 2026 Apr 2;40(6):4928–38. doi: 10.1007/s00464-026-12760-2 (PMC13672480; doi:10.1007/s00464-026-12760-2)
Supplement: Supplementary file 1 [file 464_2026_12760_MOESM1_ESM.docx]

|  | **Standardized mean difference before PSM** | **Standardized mean difference after PSM** |
| --- | --- | --- |
| **Distance** | 0.3762 | 0.0139 |
| **Sex0** | -0.2373 | 0.1207 |
| **Sex1** | 0.2373 | -0.1207 |
| **Age** | -0.0974 | 0.0820 |
| **Indication1** | -0.2376 | -0.1570 |
| **Indication2** | 0.2376 | 0.1570 |
| **BMI** | -0.0029 | 0.0906 |

***Table S1* : Standardized mean differences before and after propensity score matching (PSM). BMI: body mass index**
